# Antibiotic use in rural China: a cross-sectional survey of knowledge, attitudes and self-reported practices among caregivers in Shandong province

**DOI:** 10.1186/s12879-015-1323-z

**Published:** 2015-12-21

**Authors:** Lilu Ding, Qiang Sun, Weishuai Sun, Yihui Du, Yue Li, Xuefeng Bian, Guiqin He, Huidong Bai, Oliver J. Dyar

**Affiliations:** School of Health Care Management, Center for Health Management and Policy, Key Lab of Health Economic and Policy Research of Ministry of Health, Shandong University, Jinan, Shandong Province 250012 China; Department of Epidemiology and Health Statistics, School of Public Health, Shandong University, 44 Wenhua Xi Road, Jinan, Shandong Province 250012 People’s Republic of China; Jinan Center for Disease Control and Prevention, Jinan, Shandong Province 250021 China; Yanggu County Center for Disease Control and Prevention, Liaocheng city, Shandong Province 252300 China; Global health - Health Systems and Policy: Improving the use of medicines, Department of Public Health Sciences, Karolinska Institutet, Stockholm, 17171 Sweden

**Keywords:** Antibiotic use, KAP, Caregivers, China

## Abstract

**Background:**

To improve antibiotic use globally, we must deepen our understanding of the public’s knowledge, attitudes and practices (KAP) concerning antibiotics. Children are frequent users of antibiotics, and their caregivers play important roles in determining how antibiotics are used. The purpose of this study was to describe caregivers’ KAP in a rural province in eastern China, and to identify socio-demographic factors associated with inappropriate antibiotic use.

**Methods:**

A cross-sectional questionnaire based survey was conducted in 12 villages in one county in Shandong Province. A total of 727 individuals who were the primary day-to-day caregiver for a child aged 0–7 years were randomly selected and invited to participate. All caregivers were surveyed face-to-face using a semi-structured questionnaire focusing on the use of antibiotics in children.

**Results:**

Almost all invited caregivers (99.3 %) completed the questionnaire in full. Caregivers expressed high levels of over-expectation for antibiotics for common childhood symptoms, stating that antibiotics were always or usually necessary when a child has a fever (46 %) or dry cough (42 %). Most caregivers (93 %) were aware that they should follow the doctor’s advice when giving their children antibiotics. Many, however, reported that they had previously deviated from advice; this was most commonly through using antibiotics intermittently rather than regularly, but also by increasing and decreasing doses. Caregivers that were older and that had less formal education had higher levels of self-reported adherence (*p* < 0.01). A third of caregivers admitted to storing leftover antibiotics at home, and almost all of these caregivers (97 %) had used the antibiotics on a second occasion for their child.

**Conclusion:**

We have identified important gaps in knowledge, attitudes and practices concerning antibiotics among this rural population of caregivers. There is a clear need for multifaceted interventions that target village doctors, to improve prescribing and communication, as well as the general public, to improve health-seeking behaviours and promote responsible individual use of antibiotics.

**Electronic supplementary material:**

The online version of this article (doi:10.1186/s12879-015-1323-z) contains supplementary material, which is available to authorized users.

## Background

Antibiotics are among the most commonly used drugs worldwide, especially for young children [1]. It has been estimated that 50 % of all antibiotic prescriptions given to children are unnecessary; [2, 3] they are frequently used, for example, in children with self-limiting conditions such as upper respiratory tract infections [4–6]. Over-prescription by doctors [7], access to antibiotics without prescriptions [8, 9], and limited knowledge of caregivers [10, 11] all contribute to inappropriate antibiotic use in children worldwide. These problems are worse in low-and middle-income countries, where the prevalence of infectious diseases is higher, and fewer resources are available for maintaining hygiene, sanitation, and public health [1, 12, 13]. In addition to causing unnecessary side effects in individuals, overuse of antibiotics is the main driver for the development and spread of resistant bacteria [13–15].

In China, especially in rural areas, very little is known about the public’s knowledge and attitudes towards antibiotic use, and how these affect their practices [16]. The healthcare system in rural China consists of three tiers: county hospitals with inpatient and outpatient services, township healthcare centres with extended primary care services, and village clinics. Most villages have a village clinic for human healthcare, staffed by one or two primary care doctors with varying educational backgrounds, no support staff, and limited access to microbiology testing facilities [17]. The village clinic is the main site where rural residents seek non-emergency healthcare and can be given medications, mostly from the national essential drug list. Private drugstores are usually found in nearby towns, but not in villages. A prescription is legally required for obtaining antibiotics from a drugstore, but in practice it is not always needed in rural areas. Most rural residents participate in the New Rural Cooperative Medical Scheme of healthcare insurance. This requires all patients to pay a registration fee when visiting a doctor, and a separate fee for medications; patients receive partial reimbursement of both of these fees. Antibiotics are frequently used medications in primary care in China, with observational studies reporting that around half of all prescriptions include at least one antibiotic [18, 19]. The purpose of this study was firstly to describe i) caregivers’ understanding and expectations of antibiotic use for common childhood complaints, and ii) self-reported practices of caregivers concerning antibiotic use for their children; and secondly, to identify socio-demographic characteristics of caregivers that may contribute to inappropriate antibiotic use.

## Methods

### Study setting and design

The study was conducted in 2014 in Shandong Province in eastern China. Shandong Province consists of 17 municipalities and is subdivided into 140 counties with a total population of nearly 97 million. The income and health indicators in Shandong Province are slightly higher than the national average in China. A single county (YG) was selected within Shandong province based on the presence of local administrative support for the current study and planned interventions. YG county is a predominantly rural county, with a GDP per capita of 31,000 Yuan (≈3600 Euro in 2014) in 2013, which is slightly below average for Shandong province [20]; in other respects, it is quite typical of the province, and of rural areas in China. One township was selected at random from the 18 townships in YG county, and then twelve villages with a sufficiently large population were randomly selected from a total of 53 villages within the township. Eleven of the villages had a village clinic with one doctor, one village had two doctors. A target of 60 participants within each village was set, to produce a final sample size of 720. Local administrators created a list of all households in each village with at least one child aged 0–7 years of age (an average 8 % of total households; range 6-11 %). The village doctor in each village was asked to make a convenience sample of 60 households from the list of eligible households; an average of 118 households were eligible per village (range 82–156). In each household, an adult individual available on the day of the questionnaire was invited to participate; these participants were required to be ‘caregivers’, defined as individuals that had primary responsibility for a child aged 0–7 years old during a normal day. When a caregiver was responsible for more than one child, we collected data on the age and sex of the youngest child. The current study is part of a larger intervention project aimed at improving rural residents’ knowledge, attitudes and practices towards antibiotic use. The sample size was calculated in order to provide sufficient power for post-intervention analyses, and assumed a nonresponse rate of 5 %.

### Data collection

All selected caregivers were invited to attend a face-to-face questionnaire interview at the local village clinic on the day of the survey. The questionnaire consisted of 45 structured and semi-structured points, subdivided into four categories: (1) Demographic characteristics about the participants and their relation to the youngest child in their care; (2) Knowledge about antibiotics; (3) Attitudes towards use of antibiotics; (4) Reported practices on use of antibiotics. The questionnaire was developed based on a review of the relevant literature and in consultation with public health and infectious diseases experts. The final version included several questions that were previously asked of rural caregivers in central China in a study by Yu et al. [16]; we also assessed participants’ familiarity with commonly used antibiotics in the region, added a section on caregiver expectations for antibiotics for a range of common childhood complaints, and asked participants about intermittent and interrupted usage of antibiotics. The questionnaire was developed in Chinese and pilot tested for face validity. Graduate students from the School of Public Health, Shandong University conducted the final face-to-face interviews, and all students had received appropriate training. An English-language version of the questionnaire is included as Additional file 1.

### Data analysis

Data from the questionnaires were checked for consistency, with errors corrected by the interviewers in the field. Only fully completed questionnaires were included. Verified data were entered into a database. Statistical analysis was performed by using SPSS version 13.0 (Statistical Package for the Social Sciences Inc, Chicago, IL, USA). Categorical data were compared using the *χ*^2^ test; 95 % confidence intervals and *p*-values are presented, with a *p*-value <0.05 considered significant.

### Ethical consideration

The Ethics Committee of the School of Public Health, Shandong University, granted ethical approval for the study. Written consent forms were obtained from the participants before answering survey questions, and participants were informed that they could withdraw at any point.

## Results

### Participant characteristics

A total of 727 caregivers participated in the study, of which 722 (99.3 %) completed the questionnaire in full. Most caregivers were either mothers (44 %) or grandmothers (44.3 %) (Table 1). Most respondents under 29 years of age were parents of the child (97.5 %), whereas most respondents over 50 years of age were grandparents (97.5 %). The mean age of the children they cared for was 3.5 years (range: 0.2 to 7.0 years).

**Table 1:** Socio-demographic characteristics of the 722 caregivers

Socio-demographic characteristics	Number (n)	Percentage (%)
Sex		
Male	70	9.7
Female	652	90.3
Age (years)		
≤ 29	158	21.9
30–49	284	39.3
≥ 50	280	38.8
Education level		
Primary school and below	198	41.3
Junior school	304	42.1
Senior school and above	120	16.6
Annual household income (Yuan)		
< 10,000 [<1200 Euro]	78	10.8
10,000–30,000 [1200–3600 Euro]	384	53.2
> 30,000 [>3600 Euro]	260	36.0
Relation		
Parents	356	49.3
Grandparents	351	48.6
Other	15	2.1
Number of children		
1	659	91.3
2	60	8.3
3	3	0.4
Age of child (years)		
< 3	289	40.0
≥ 3	433	60.0
Gender of child		
Male	432	59.8
Female	290	40.2

### Knowledge and attitudes towards use of antibiotics

**Fig. 1:**
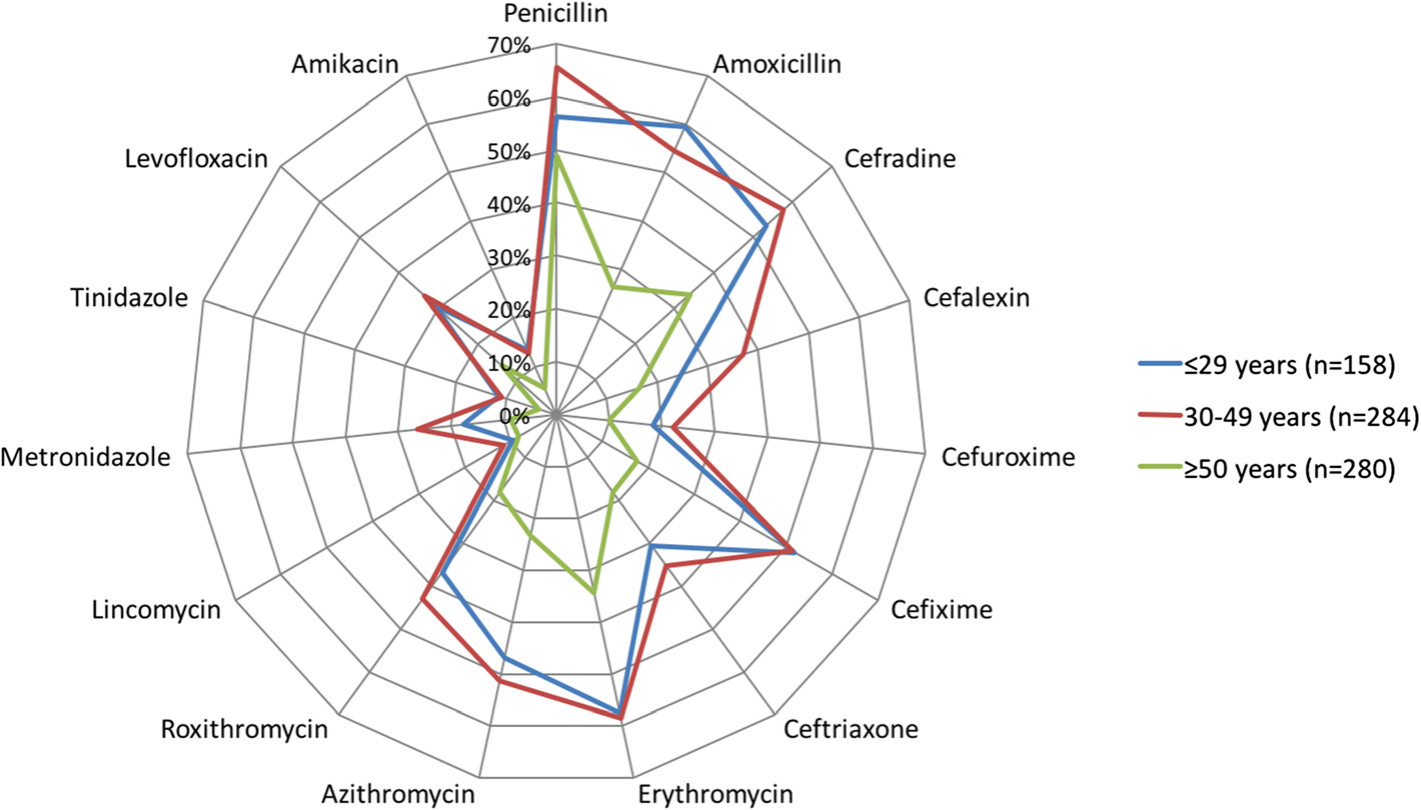
Recognition of specific antibiotics**.** Percentage of participants who correctly identified each medication as an antibiotic, shown by participant age group

Participants were shown a list of fifteen of the most commonly used antibiotics in rural Shandong, as determined in a previous study [21]. Each participant was asked if they had heard of the medication before. If they had, they were then asked whether they thought the medication was an antibiotic or not. Overall 14 (1.94 %) of the participants correctly identified all the medications as antibiotics. Younger participants more frequently recognized each antibiotic as being an antibiotic, compared with older participants (Fig. 1), and the most commonly recognized antibiotics were penicillin (57.0 %), erythromycin (48.9 %), cefradine (47.5 %) and amoxicillin (44.7 %). A small number of participants (4.4 %) were unable to recognize any antibiotic.

Almost all participants (668/722, 92.5 %) were aware that they should follow the instructions given by the doctor when taking antibiotics. Over half of the participants (452/722, 62.6 %) were aware that a prescription was needed to get antibiotics, and 56.5 % (408/722) said they knew that inappropriate antibiotic use could add to the risk of antibiotic resistance. Participants who had an educational level higher than primary school, who were aged over 30, or who were grandparents, were all more likely to be aware that a prescription was needed to get antibiotics, and that inappropriate antibiotic use can add to the risk of antibiotic resistance (*p <* 0.01 for all). The main sources of information about antibiotic use were doctors (85 %) and television (33 %). Participants with an education level of high school or above were more likely to obtain information about antibiotic use from books or magazines (15 vs. 4.8 %, *p* < 0.001), and the internet (21.7 vs. 6.8 %, *p* < 0.001), compared with participants with a lower education level.

Many participants (38.4 %) agreed that antibiotics are used too much in China, a similar proportion were uncertain (37.8 %), and some disagreed (23.8 %). Forty-one percent of participants agreed that it is dangerous for children to become infected with antibiotic-resistant bacteria, whereas 12.9 % disagreed and 46.1 % were uncertain.

Participants were asked how often antibiotics are needed for children when they have a variety of common symptoms and conditions. Table 2 summarizes their responses, with over-expectation for an antibiotic defined as answering ‘Always’ or ‘Usually’. The highest levels over-expectation for an antibiotic were observed for fever (46.4 %), dry cough (42.3 %) and bronchitis (38.1 %).

**Table 2:** Participants’ beliefs about requirements for an antibiotic for common childhood complaints (*n* = 722)

How often an antibiotic is needed for a child with this complaint	Bronchitis	Diarrhea	Ear infection	Fever	Dry cough	Sore throat	Stuffy nose
Always	23.7 %	16.3 %	16.8 %	29.8 %	26.0 %	23.1 %	19.1 %
Usually	14.4 %	10.5 %	7.1 %	16.6 %	16.3 %	13.3 %	13.0 %
Sometimes	19.7 %	17.0 %	13.0 %	16.6 %	14.4 %	15.7 %	17.6 %
Never	7.8 %	24.2 %	8.2 %	13.4 %	18.6 %	18.6 %	22.2 %
Don’t know	34.5 %	31.9 %	55.0 %	23.5 %	24.7 %	29.4 %	28.1 %
Over-expectation for an antibiotic	38.1 %	26.8 %	23.9 %	46.4 %	42.3 %	36.4 %	32.1 %

Based on their responses to antibiotic needs for common complaints, participants were divided into a ‘high’ over-expectation group (over-expectation for an antibiotic for three or more complaints, *n* = 335) and a ‘low’ over-expectation group (over-expectation for an antibiotic for two or fewer complaints, *n* = 387). Table 3 shows the association of different participant socio-demographic factors and levels of over-expectation. Being the caregiver for a child over the age of three was the only factor significantly associated with higher levels of over-expectation for antibiotics.

**Table 3:** Relation of socio-demographic characteristics with over-expectation for antibiotics for common childhood complaints

Socio-demographic factors	High over-expectation (expectation for an antibiotic for 3 or more complaints)	Low over-expectation (expectation for an antibiotic for 2 or fewer complaints)	*p*-value
	*n* (%)	*n* (%)	
Age (years)			
≤ 29	68 (43.0)	90 (57.0)	0.407
30 ~ 49	140 (49.3)	144 (50.7)	
≥ 50	127 (45.4)	153 (54.6)	
Sex			
Male	29 (41.4)	41 (58.6)	0.380
Female	306 (46.9)	346 (53.1)	
Educational level			
Primary school and below	133 (44.6)	165 (55.4)	0.484
Junior school	149 (49.0)	155 (51.0)	
Senior school and above	53 (44.2)	67 (55.8)	
Household income level (Yuan)			
< 10,000 [<1200 Euro]	36 (46.2)	42 (53.8)	0.462
10,000–30,000 [1200–3600 Euro]	186 (48.4)	198 (51.6)	
> 30,000 [>3600 Euro]	113 (43.5)	147 (56.5)	
Relation with child			
Parents	167 (46.9)	189 (53.1)	0.810
Grandparents	160 (45.6)	191 (54.4)	
Other	8 (53.3)	7 (46.7)	
Number of child			
1	306 (46.4)	353 (53.6)	0.762
2	27 (45.0)	33 (55.0)	
3	2 (66.7)	1 (33.3)	
Age of child (years)			
< 3	114 (39.4)	175 (60.6)	0.02
≥ 3	221 (51.0)	212 (49.0)	
Sex of child			
Male	208 (48.1)	224 (51.9)	0.250
Female	127(43.8)	163(56.2)	

### Reported antibiotic use for children

Almost a third of participants (31.6 %, 228/722) said that they stored antibiotics at home for their children, and a further 16.2 % (117/722) stored medicines for their children but were unsure if they were antibiotics or not. Almost all of the participants who admitted to storing antibiotics at home for their children reported that they had used them on a second occasion (97.4 %, 211/228), with 39.5 % always or usually using stored antibiotics when their child became sick. Participants had most frequently obtained these stored antibiotics from the healthcare facilities such as the village clinic (68.9 %, 157/228) and township or county hospitals (10.5 %, 24/228). A fifth of participants obtained the antibiotics from drugstores (20.6 %, 47/228). Participants who knew that a prescription was needed to get an antibiotic were more likely to report storing leftover antibiotics at home (36.3 % vs. 23.7 %, *p* < 0.01).

Participants were asked about how they had used antibiotics for their children in the past, including how often they had followed the doctor’s instructions. Table 4 summarizes these self-reported behaviours, with the most common deviation from a doctor’s advice being to use antibiotics intermittently or in an interrupted pattern (reported by 45 %). We categorized participants into ‘high adherence’ (four or more high adherence answers, *n* = 520) and ‘low adherence’ (three or fewer high adherence answers, *n* = 202) groups based on these responses, which indicated how frequently participants would deviate from a doctor’s advice on how to use antibiotics for their children. Table 5 shows the association of different socio-demographic characteristics with levels of adherence. Finally, participants were asked when they normally stop giving antibiotics to their children; 48 % reported that they usually or always stopped as soon as symptoms of an illness disappeared.

**Table 4:** Ratings of participants’ compliance with doctors’ directions on antibiotic use (*n* = 722)

Antibiotic use practices	Always	Usually	Sometimes	Seldom	Never
How often do you use antibiotics prophylactically, to protect your child from diseases like the common cold?	1.0 %	1.9 %	3.2 %	4.6 %	**89.3 %**
How often do you follow doctors’ instructions when giving your child antibiotics?	**82.8 %**	10.8 %	5.8 %	0.4 %	0.1 %
Have you ever increased the dosage of antibiotics for your child for better efficacy?	0.6 %	1.2 %	1.9 %	6.1 %	**90.2 %**
Have you ever reduced the dosage of antibiotics for your child for improved safety?	3.2 %	3.6 %	5.4 %	11.5 %	**76.3 %**
Have you ever used antibiotics intermittently or in an interrupted pattern for your child?	1.4 %	4.8 %	6.4 %	32.7 %	**54.7 %**

Numbers in bold are the ‘high adherence’ answers for each question

**Table 5:** Association between participants’ socio-demographic characteristics and behaviours associated with adherence

Socio-demographic factors	Low adherence (3 or fewer high adherence answers)	High adherence (4 or more high adherence answers)	*p*-value
	*n* (%)	*n* (%)	
Age (years)			
≤ 29	64 (40.5)	94 (59.5)	0.000
30 ~ 49	81 (28.5)	203 (71.5)	
≥ 50	57 (20.4)	223 (79.6)	
Sex			
Male	12 (17.1)	58 (82.9)	0.034
Female	190 (29.1)	462 (70.9)	
Educational level			
Primary school and below	68 (22.8)	230 (77.2)	0.003
Junior school	87 (28.6)	217 (71.4)	
Senior school and above	47 (39.2)	73 (60.8)	
Household income level (Yuan)			
< 10,000	15 (19.2)	63 (80.8)	0.189
10,000 ~ 30,000	111 (28.9)	273 (71.1)	
> 30,000	76 (29.2)	184 (70.8)	
Relation with child			
Parents	128 (36.0)	228 (64.0)	0.000
Grandparents	70 (19.9)	281 (80.1)	
Other	4 (26.7)	11 (73.3)	
Number of child			
1	182 (27.6)	477 (72.4)	0.783
2	19 (31.7)	41 (68.3)	
3	1 (33.3)	2 (66.7)	
Age of child (years)			
< 3	86 (29.8)	203 (70.2)	0.384
≥ 3	116 (26.8)	317 (73.2)	
Sex of child			
Male	122 (28.2)	310 (71.8)	0.848
Female	80 (27.6)	210 (72.4)	

## Discussion

We have surveyed over 700 adults living in villages in a province in eastern China about their knowledge, attitudes and practices concerning antibiotics in children. Each participant is the primary caregiver for a child under the age of 7, a demographic commonly associated with high use of antibiotics. Despite limiting our investigation to this single specific demographic, our results indicate that multifaceted and multi-target interventions are vital to improve antibiotic use in rural China. To the best of our knowledge, this is only the second ever study of this type conducted among caregivers in China, a country in which a fifth of the world’s population resides. Several of the results from our study are similar to those of Yu et al.*,* conducted among rural caregivers attending vaccination clinics in central China [16]. This similarity indicates that interventions aimed at improving antibiotic practices need to be applied over a wide population base and geographical area; these interventions must take into account the growing socio-demographic heterogeneity in the rural population, as young parents more frequently travel to nearby towns for work during the weekdays, and children are left in the care of grandparents.

The village doctor plays a central role in determining antibiotic use: 70 % of antibiotics that participants admitted to having stored in their homes were obtained from village clinics, and the village doctor was, by a wide margin, the most frequently mentioned source of information about antibiotic use, as was also found among rural caregivers in central China [16]. Almost all participants (93 %) knew they should follow the doctor’s advice about taking antibiotics. All of these results indicate the need for a well-trained base of village doctors, capable not only of determining when antibiotics are needed, but also describing how to use them once they have been prescribed; as Yu et al. reported, village doctors frequently do not explain a condition or treatment to caregivers, but those who had received explanations were more likely to follow them [16]. This need becomes more important, and also more influential, in the context of policies aimed at reducing illegal over the counter dispensing of antibiotics [22, 23]. It is encouraging to see that in a recent study in another part of Shandong province over 90 % of village clinic doctors had accessed training on antibiotic use, and most had done this in the previous three years [24]; however, even though only 5 % of these village doctors said they would use antibiotics for a patient with common cold, a prescription analysis at the same village clinics found that over half of common cold prescriptions included at least one antibiotic. In a qualitative study in another rural province, Reynolds et al. found that doctors used antibiotics to speed recovery and respond to patient expectations, even if they did not believe antibiotics were needed to treat an infection [8].

Although participants view the doctor’s advice on use of antibiotics as important, only 83 % of participants reported that they always follow this advice. On further questioning, many participants admitted that they had made adjustments in antibiotic use, such as choosing to lower a dose for efficacy, and almost half said that they had on a previous occasion given their children an antibiotic intermittently rather than regularly as prescribed. In addition, close to a half of participants reported always or sometimes stopping antibiotics once symptoms started to improve, as did 63 % of rural caregivers in central China [16]. All of these behaviours can easily lead to under-dosing, with risks of treatment failure and selection of resistant bacteria [25]. Participants that were older and that had lower levels of education were less likely to report deviating from a doctor’s advice; however, it is possible that these individuals were expressing a greater degree of reliance on doctor’s advice, rather than purposeful adherence.

Nearly two-thirds of participants knew that a prescription was needed to obtain antibiotics. At the same time, a third of participants admitted that they stored antibiotics at home for their children, and almost all had used these antibiotics on a second occasion for their child. It appears that the need for a prescription for antibiotics is not a strong enough signal that such drugs should only be used with input from a trained healthcare professional – indeed, participants who knew that a prescription was needed to get antibiotics were actually more likely to store leftover antibiotics at home. In the study by Yu et al., rural caregivers who did not know that a prescription was needed to get antibiotics were more likely to have purchased antibiotics at a drugstore without a prescription [16]; here we have found that awareness of the need for a prescription alone may change behaviour, but it may not improve how antibiotics are actually used. Fixed pack dispensing, which is not currently widely practiced in China, may be a complementary method to help reduce the availability of leftover antibiotics in homes [26].

We found gaps in participant’s knowledge about antibiotic use, and community focused interventions may have an important role to play in addressing these [27]. Firstly, some participants are probably not aware what an antibiotic is. Using a list of the fifteen most commonly used antibiotics in the area, 4.4 % of people were still unable to identify a single medication as being an antibiotic. Secondly, there was very little awareness about how often antibiotics are truly needed for a variety of common childhood symptoms and illnesses. Between 23.5 % and 55 % of participants responded that they did not know whether antibiotics were needed for each complaint; and close to a half of all participants suggested that a fever or a dry cough should always or usually be treated with antibiotics; similarly, 34 % of rural caregivers in central China reported that antibiotics should always be used when a child has a fever [16]. Meanwhile, some participants thought that antibiotics were never necessary for a fever (13.4 %) or a sore throat (18.6 %). Over-expectation, under-expectation and a lack of knowledge all co-exist among caregivers in this setting. We have not investigated to what extent these expectations direct health-seeking behaviours, but they have the potential to lead to both problems of inappropriate overuse and underuse of antibiotics [1, 16]. The only socio-demographic factor associated with high levels of over-expectation was having a child over the age of three; a possible explanation for this is that the participants’ expectations are driven by experience, rather than knowledge, with caregivers of older children having had more exposure to antibiotics being used for their children, and so they become more likely to think this is normal. Thirdly, just over a half of respondents knew that inappropriate antibiotic usage adds to the risk of antibiotic resistance. This is the same proportion as reported in rural caregivers in central China concerning excessive antibiotic use (57 %) [16], and slightly lower than the median 70 % reported in a recent global systematic review of the general public’s knowledge and attitudes towards antibiotic resistance [28]. Furthermore, only 38 % of participants agreed that antibiotics were overused in China, and most participants did not think that it was dangerous for their children to be infected with antibiotic-resistant bacteria. This lack of awareness may contribute to the widespread behaviours of participants storing and deciding to use antibiotics by themselves, and to adjusting antibiotic dosing without asking the doctor for advice. A three year national campaign on antibiotic resistance was launched by the Ministry of Health in 2011, but focussed mainly on improving antibiotic use in secondary and tertiary care hospitals in China [29]. Although there were no major public education elements to the campaign, there have recently been accounts of the problems of antibiotic resistance on mass media, which will likely have promoted public awareness of the issue.

Our study has several strengths, including a high response rate with a randomized selection of a large population of caregivers from twelve different villages, and the use of trained interviewers to conduct the questionnaires. We also have important limitations: first, we were only able to select villages with at least 60 households with children aged 0–7, in order to include a sufficient number of participants for the planned educational intervention; this means that the selected villages were sampled from the largest 40 % of villages in the township, but are similar in other respects to all villages in the study setting. Secondly, a convenience sample was used to select the final participants; however, a large proportion of eligible households within each village were included (39-73 %), and we have no reason to suspect that it led to the introduction of any major biases. Thirdly, participants were primary caregivers for their children, but this does not mean that they take full responsibility for decision-making when children under their care are ill. Fourthly, our study investigated reported rather than observed practices, and we did not gather any data on actual frequency of antibiotic use in children. Overall, the consistency of several of our findings with those of Yu et al. in central China [16] suggest that many of our conclusions may be generalizable to caregivers living in villages in other parts of rural China.

## Conclusions

We have identified important gaps between knowledge and reported practices among caregivers in rural China in the context of antibiotic use for the children they care for. Our findings reinforce the need for multifaceted interventions to improve antibiotic use, and provide suggestions for how educational approaches could be targeted. We have used these results to develop an intervention for caregivers in the study area with the aim of improving knowledge, attitudes and practices. This is led by a paediatrician from a local hospital, and involves regular seminars in the villages on health-seeking behaviours and responsible antibiotic use, and posters in the village clinics. In a separate project we are also working directly with village doctors to improve antibiotic prescribing in another part of Shandong province. Further studies are needed to provide data on actual rather than reported behaviour among caregivers, and to more clearly delineate the role that village doctors play in setting expectations for antibiotic use among caregivers.

## Additional file

Additional file 1:
**Research questionnaire of knowledge, attitudes and practices of antibiotics among Caregivers in the rural area of Shandong Province.** (DOCX 31 kb)
