# Supplementary material for: Antibiotic use in rural China: a cross-sectional survey of knowledge, attitudes and self-reported practices among caregivers in Shandong province
Source: BMC Infect Dis. 2015 Dec 21;15:576. doi: 10.1186/s12879-015-1323-z (PMC4687375; doi:10.1186/s12879-015-1323-z)
Supplement: Additional file 1: — Research questionnaire of knowledge, attitudes and practices of antibiotics among Caregivers in the rural area of Shandong Province. (DOCX 31 kb) [file 12879_2015_1323_MOESM1_ESM.docx]

Research questionnaire of knowledge, attitudes and practices of antibiotics among Caregivers in the rural area of Shandong Province

Village: ____________ Name: ____________

No.：___________ Date of investigation: _____Year____Month____Day

Investigator：_________

Examinator：________

**Demographic characteristics**

| 1 | Sex: ①Male ②Female |  |
| --- | --- | --- |
| 2 | Age: years old |  |
| 3 | Your educational status:  ① Illiterate ② Primary School ③ Secondary School ④High School/ Secondary technical college ⑤College or above |  |
| 4 | Relationship with your child：  ① Father ② Mother ③ Grandfather ④ Grandmother ⑤other |  |
| 5 | Who is mainly taking care the child?  ① Father ② Mother ③ Grandfather ④ Grandmother ⑤other |  |
| 5.1 | In case your child gets a cold or fever, who will decide whether to take the child to see a doctor?  ① Father ② Mother ③ Grandfather ④ Grandmother ⑤other |  |
| 5.2 | How much can you decide how to take care the child when he/she get sick?  Rating on a scale from 0 (no control) to 10 (full control) |  |
| 6 | Your family income in 2013？ Yuan |  |
| 6.1 | How much money can you get from the child’s parents? Yuan (grandparents answer this question) |  |
| 7 | Number of household members？ |  |
| 8 | Number of children aged 0～7 years old : |  |
|  | General condition of children(asking for the information of the youngest child, if there are more than one children) | |
| 9 | Age years old |  |
| 10 | Sex ① Male ② Female |  |
| 11 | How many times did the child suffer from a disease in the past 6 months.? |  |
| 12 | What diseases did the child have? |  |
| 13 | What were the main medical institutions you choose for your child?  ① Village clinic ② Township health center ③ County level hospital ④ Private drug store ⑤ Other |  |
| 14 | What do you think of the child's health status?  Rating on a scale from 0 (extremely poor) to 100 (extremely good) |  |

**Section A Knowledge**

| 15 Name of medication | | Have you ever heard of this medication before?  ①yes  ②no | Do you think this is antibiotic?  ①yes ②no  ③don’t know | name of medication | Have you ever heard of this medication before?  ①yes  ②no | Do you think this is antibiotic?  ①yes ②no  ③don’t know | name of medication | Have you ever heard of this medication before?  ①yes  ②no | Do you think this is antibiotic?  ①yes ②no  ③don’t know |
| --- | --- | --- | --- | --- | --- | --- | --- | --- | --- |
| Ceftriaxone | |  |  | Amoxicillin |  |  | Penicillin |  |  |
| Cefradine | |  |  | Azithromycin |  |  | Amikacin |  |  |
| Cefalexin | |  |  | Lincomycin |  |  | Metronidazole |  |  |
| Cefuroxime | |  |  | Erythromycin |  |  | Tinidazole |  |  |
| Cefixime | |  |  | Roxithromycin |  |  | Levofloxacin |  |  |
| 16 | Are you aware that you should follow the instructions given by the doctor when taking antibiotics?  ①yes ②no | | | | | | | |  |
| 17 | Do you know that inappropriate antibiotic use could add to the risk of antibiotic resistance?  ①yes ②no | | | | | | | |  |
| 18 | Are you aware that a prescription is needed to get antibiotics? ①yes ②no | | | | | | | |  |
| 19 | What are the main sources of information about antibiotic use? (can be multiple)  ①doctors ②seminar ③drug store ④television ⑤internet ⑥books or magazines ⑦antibiotic brochure  ⑧other | | | | | | | |  |

**Section B Attitudes**

| 20 | Prophylactic use of antibiotic can prevent some diseases (like common cold)  ①agree ②disagree ③Don't know | |  |
| --- | --- | --- | --- |
| 21 | The use of antibiotics is too much in China  ①agree ②disagree ③Don't know | |  |
| 22 | It is dangerous for children to become infected with antibiotic-resistant bacteria  ①agree ②disagree ③Don't know | |  |
| 23 | The use of combination antibiotics has better effects  ①agree ②disagree ③Don't know | |  |
| 24 | One can stop taking a full course of antibiotic if the symptoms are improving  ①agree ②disagree ③Don't know | |  |
| 25 | Antibiotics with higher prices have better effects  ①agree ②disagree ③Don't know | |  |
| 26 | Would you take the initiative to request the doctor to prescribe antibiotics?  ① Yes ② No | |  |
| 27 | Do you think it should be necessary to have a prescription to purchase antibiotics?  ①necessary ②unnecessary ②Don't know | |  |
|  | Please let us know your attitude towards the need to use antibiotics if your child is suffering from the following diseases: | | |
| 28 | Bronchitis | ①Always ②Usually ③Sometimes ④Never ⑤Don't know |  |
| 29 | Diarrhea | ①Always ②Usually ③Sometimes ④Never ⑤Don't know |  |
| 30 | Ear Infection | ①Always ②Usually ③Sometimes ④Never ⑤Don't know |  |
| 31 | Fever | ①Always ②Usually ③Sometimes ④Never ⑤Don't know |  |
| 32 | Dry Cough | ①Always ②Usually ③Sometimes ④Never ⑤Don't know |  |
| 33 | Sore Throat | ①Always ②Usually ③Sometimes ④Never ⑤Don't know |  |
| 34 | Stuffy Nose | ①Always ②Usually ③Sometimes ④Never ⑤Don't know |  |

**Section C Self-reported Practices**

| 35 | Do you store antibiotics at home for your children?  ①yes ②no ③unsure if they were antibiotics or not. |  |  |  |
| --- | --- | --- | --- | --- |
| 35.1 | Please tell us the name of the medication you stored? |  |  |  |
| 35.2 | Where did you get these medications? (can be multiple)  ①village clinic ②township health center ③county level hospital ④private drug store ⑤other |  |  |  |
| 35.3 | How often do you use stored antibiotics when a child becomes sick again?  ①Always ②Usually ③Sometimes ④Seldom ⑤Never |  |  |  |
| 36 | How often do you use antibiotics prophylactically, to protect your child from diseases like the common cold?  ①Always ②Usually ③Sometimes ④Seldom ⑤Never |  |  |  |
| 37 | How often do you follow doctors’ instructions when giving your child antibiotics?  ①Always ②Usually ③Sometimes ④Seldom ⑤Never |  |  |  |
| 38 | Have you ever increased the dosage of antibiotics for your child for better efficacy? ①Always ②Usually ③Sometimes ④Seldom ⑤Never |  |  |  |
| 39 | Have you ever reduced the dosage of antibiotics for your child for improved safety? ①Always ②Usually ③Sometimes ④Seldom ⑤Never |  |  |  |
| 40 | Have you ever used more than one antibiotic at a time for your child?  ①Always ②Usually ③Sometimes ④Seldom ⑤Never |  |  |  |
| 41 | Have you ever used antibiotics intermittently or in an interrupted pattern for your child?  ①Always ②Usually ③Sometimes ④Seldom ⑤Never |  |  |  |
| 42 | Have you ever withdrawn antibiotics when the symptoms improved for your child?  ①Always ②Usually ③Sometimes ④Seldom ⑤Never |  |  |  |
| 43 | Have you ever withdrawn antibiotics when the symptoms disappeared for your child?  ①Always ②Usually ③Sometimes ④Seldom ⑤Never |  |  |  |
| 44 | Have you ever continued the use of antibiotics for your child when the symptoms have disappeared?  ①Always ②Usually ③Sometimes ④Seldom ⑤Never |  |  |  |
| 45 | What factors do you consider when you choose antibiotics?  ①Price ②Brand ③Doctors’ advices ④Other people’s advices | | |  |
